# Regional Prediction of Ozone and Fine Particulate Matter Using Diffusion Convolutional Recurrent Neural Network

**DOI:** 10.3390/ijerph19073988

**Published:** 2022-03-27

**Authors:** Dongsheng Wang, Hong-Wei Wang, Kai-Fa Lu, Zhong-Ren Peng, Juanhao Zhao

**Affiliations:** 1 Center for Intelligent Transportation Systems and Unmanned Aerial Systems Applications Research, State Key Laboratory of Ocean Engineering, School of Naval Architecture, Ocean and Civil Engineering, Shanghai Jiao Tong University, Shanghai 200240, China; dongshengwang_sjtu@163.com; 2 International Center for Adaptation Planning and Design, College of Design, Construction and Planning, University of Florida, P.O. Box 115706, Gainesville, FL 32611, USA; kaifa.lu@ufl.edu; 3 Department of Computer Science, Viterbi School of Engineering, University of Southern California, Los Angeles, CA 90089, USA; juanhaoz@usc.edu

**Keywords:** fine particulate matter, ozone, air quality forecast, diffusion convolutional recurrent neural network, deep learning

## Abstract

Accurate air quality forecasts can provide data-driven supports for governmental departments to control air pollution and further protect the health of residents. However, existing air quality forecasting models mainly focus on site-specific time series forecasts at a local level, and rarely consider the spatiotemporal relationships among regional monitoring stations. As a novelty, we construct a diffusion convolutional recurrent neural network (DCRNN) model that fully considers the influence of geographic distance and dominant wind direction on the regional variations in air quality through different combinations of directed and undirected graphs. The hourly fine particulate matter (PM_2.5_) and ozone data from 123 air quality monitoring stations in the Yangtze River Delta, China are used to evaluate the performance of the DCRNN model in the regional prediction of PM_2.5_ and ozone concentrations. Results show that the proposed DCRNN model outperforms the baseline models in prediction accuracy. Compared with the undirected graph model, the directed graph model considering the effects of wind direction performs better in 24 h predictions of pollutant concentrations. In addition, more accurate forecasts of both PM_2.5_ and ozone are found at a regional level where monitoring stations are distributed densely rather than sparsely. Therefore, the proposed model can assist environmental researchers to further improve the technologies of air quality forecasts and could also serve as tools for environmental policymakers to implement pollution control measures.

## 1. Introduction

In recent decades, developing countries such as China have experienced rapid economic growth and urbanization, and the substantial problems of urban air pollution have also emerged [1,2]. For example, frequent occurrences of haze weather have attracted worldwide concerns due to the deterioration of urban particulate pollution closely related to intensive emissions of fine particles (PM_2.5_) and coarse particles (PM_10_). According to epidemiological studies, long-term exposure to higher concentration levels of particulate matter (PM) can cause serious health risks, such as cardiovascular diseases, respiratory diseases, and even deaths [3,4]. Additionally, PM-related visibility reduction also brings negative effects on human production and daily life [5]. Tropospheric ozone is another air pollutant and one of the most important greenhouse gases. Ozone can participate in various atmospheric photochemical processes and contribute to the indirect formation of secondary particulate matter, which also exhibits very detrimental effects on human health [6,7]. Therefore, the accurate prediction of particulate matter and ozone in a high spatial and temporal resolution is essential in assisting governmental departments to design and implement the related emission and pollution control policies, and the refined regional forecasts of PM and ozone concentrations could strongly enhance the protection of public health.

Related research on air quality forecasts has mainly been conducted with deterministic models and empirical models. The deterministic models are developed based on atmospheric physics and mechanisms, such as the Weather Research and Forecasting model coupled with Chemistry (WRF-Chem) [8,9] and Community Multi-scale Air Quality (CMAQ) model [10]. The models can help researchers understand the physical and chemical formation mechanisms of urban air pollution but require lots of parameters including meteorological conditions and emission inventories as inputs, thus bringing high uncertainty in air quality forecasts. Furthermore, emission inventories may change over time and need to be updated from time to time, which are usually accompanied by larger amounts of costs and difficulties. By contrast, the empirical models are built based on statistics and machine learning approaches. Although the models could not deeply explore the meteorological and chemical coupling patterns behind air pollution, they often only require a few extra parameters as model inputs in addition to historical data of air pollutants. Hence, statistical methods and machine learning models are widely used to achieve the time series predictions of urban air pollution, such as Auto-regressive Integrated Moving Average (ARIMA) [11], Support Vector Machine (SVM) [12], Classification and Regression Tree (CART) [13], etc. These models could fully cover different types of inputs including air pollutants, meteorology, land use, etc., but the spatial characteristics of air pollution data cannot be sufficiently incorporated in the process of model construction.

Deep learning models have presented excellent performances in the fields of air quality forecasts and environmental assessments, such as the Recurrent Neural Network (RNN) [14], Long Short-Term Memory (LSTM) [15,16,17,18,19], and Gated Recurrent Unit (GRU) [20,21]. However, these RNN-based models cannot fully consider the physical characteristics of topology networks among air quality monitoring stations to characterize the spatial correlations [22]. Motivated by the potentials of the Convolutional Neural Network (CNN) in capturing the spatial relationships, CNN-LSTM [22] and Convolutional LSTM [23] are employed to conduct air quality predictions by extracting both spatial and temporal features in grid-structured data (e.g., images). However, the CNN-based models only extract the spatial features of the research target areas from the input grid-structured data and are incapable of modeling complex topological relationships among large-scale air quality monitoring network. The graph neural network (GNN) is an emerging deep learning model that could map the complex topological relationships of certain spatial areas into a low-dimensional matrix. With this potential, the GNN have been widely applied in air quality forecast tasks [24,25], and outperforms the common deep learning models mentioned above. Generally, these GNN models [26] use undirected graphs to capture the topological relationships among air quality monitoring network, but the undirected edges between nodes cannot consider the effects of wind direction in the graph. Considering that air pollutants can be transported following the direction of the wind, an integration of the wind factors into the GNN model may improve the prediction performance. Thus, an exploration of the GNN model driven by a directed graph is necessary to model the influence of wind factors and further improve regional air quality prediction.

To fill the above research gaps, we develop a novel diffusion convolutional recurrent neural network (DCRNN) model for the regional prediction of PM_2.5_ and ozone concentrations. Specifically, different graph construction methods including undirected and directed graphs are separately integrated into the proposed DCRNN model to fully consider the spatial relationships among air quality monitoring stations. The main difference between undirected and directed graphs lies in that the directed graph considers the network-level dominant wind direction as an extra and important spatial dependency among monitoring stations while the undirected graph does not. Then, we further evaluate the performances of different DCRNN models in forecasting the PM_2.5_ and ozone concentrations and compare them with baseline models for various prediction lengths of time. Finally, we discuss the influence of spatial characteristics within the proposed DCRNN model on the accuracy of air quality forecasts.

## 2. Data and Methods

### 2.1. Data Description

**Figure 1:**
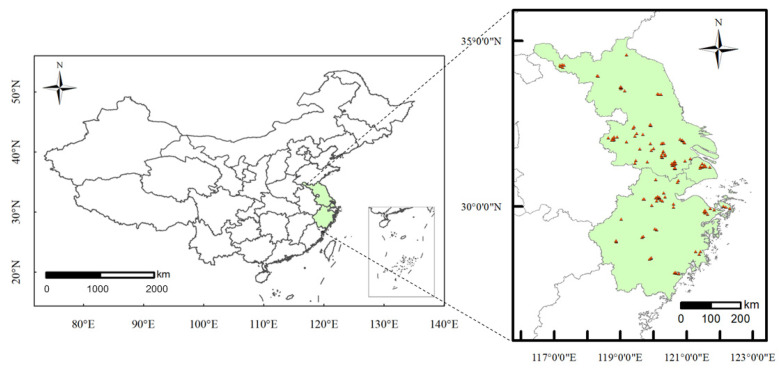
Geographical location of the study area and spatial distributions of 123 air quality monitoring stations.

The Yangtze River Delta region is selected as the study area covering three provincial units (i.e., Zhejiang, Jiangsu, and Shanghai), and its latitude and longitude range from 27° N to 35° N and 116° E to 123° E, respectively. Figure 1 shows the geographical location of the study area and the distributions of 123 air quality monitoring stations. In this study, hourly data of six air pollutants (e.g., PM_2.5_, O_3_, PM_10_, SO_2_, NO_2_, and CO) from monitoring stations between January 2015 and December 2018 are used to feed the proposed deep learning model. Hourly grid-level weather data (e.g., temperature, humidity, air pressure, precipitation, and wind speed at both X- and Y-axis) during the same period are generated by the Weather Research and Forecasting (WRF) model with a grid resolution of 5 km × 5 km, as extra model inputs. For convenience of graph construction, the air pollutant and meteorology datasets are divided into two groups according to seasonal discrepancies: one group contains the summer and autumn data from April to September (higher ozone and lower PM_2.5_ concentrations) and the other group includes the winter and spring data from October to March (lower ozone and higher PM_2.5_ concentrations).

### 2.2. Diffusion Convolution

The Convolutional Neural Network (CNN) is a widely-used network structure that uses the filters containing convolutional kernels to extract spatial features from grid-structured data such as images. With this motivation, a similar idea can be extended into the graph-structured data to extract spatial features from the data and build a model, which is also the essential idea of diffusion convolution.

**Figure 2:**
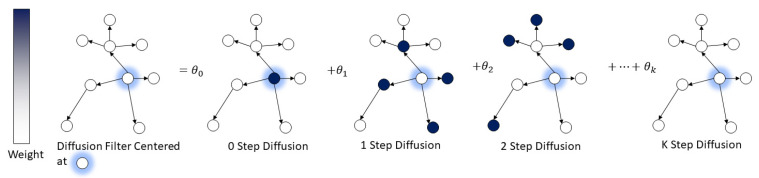
Illustration of the diffusion convolution process with *K* diffusion steps.

Diffusion convolution [24] is defined as a combination of the diffusion processes with different steps over the graph. Specifically, the *K* diffusion steps represent the distance of each node in the graph from the current forecasting position, i.e., how many edges are passed to reach the center point, as shown in Figure 2. For each node, the model calculates the neighbors from 0 to *k* steps away from itself separately and computes the corresponding transition matrix for each step. The probability θ is a learnable parameter to combine all transition matrices into a diffusion convolution filter when training the model. Here, the diffusion convolution operator ★B over a graph signal $X\in {\mathbb{R}}^{I\times N}$ and the filter $f_{\theta }$ are defined as:

(1) $$X_{:,\;n}★Bf_{\theta }=\sum_{k=0}^{K-1}\left(\theta_{k,1}{\left(D_{up}^{-1}A\right)}^{k}+\theta_{k,2}{\left(D_{down}^{-1}A^{T}\right)}^{k}\right)X_{:,\;n\;}\;\mathrm{for}\;n\in \left\{0,\ldots ,N\right\}$$

where $\theta \in {\mathbb{R}}^{K\times 2}$ is the probability parameter for the filter, $D_{up}$ and $D_{down}$ represent the in-degree and out-degree diagonal matrix of a graph, and $D_{up}^{-1}A$ and $D_{down}^{-1}A^{T}$ represent the transition matrices of the forward and backward diffusion processes, respectively. Particularly, for undirected graphs, $D_{up}$ is equal to $D_{down}$, and $D_{up}^{-1}A$ is also equal to $D_{down}^{-1}A^{T}$. With the diffusion convolution filter and activation function, the diffusion convolution layer in a neural network can map N-dimensional features to M-dimensional outputs.

### 2.3. Diffusion Convolutional Recurrent Neural Network (DCRNN)

The DCRNN model captures the spatial dependencies extracted by diffusion convolution and integrates them into the Recurrent Neural Network (RNN) model to handle time series data. The basic principle of RNN is to consider the current inputs as hidden states and process the information from the previous inputs with a multi-gate mechanism [27]. Specifically, the initial GRU sets two internal gated recurrent units to capture the long-term dependencies from time series data. The gate signals of GRU are first computed as follows:

(2) $$r^{\left(t\right)}=\delta \left(W_{xr}X^{\left(t\right)}+W_{hr}H^{\left(t-1\right)}+b_{r}\right)$$

(3) $$z^{\left(t\right)}=\delta \left(W_{xz}X^{\left(t\right)}+W_{hz}H^{\left(t-1\right)}+b_{z}\right)$$

where $r^{\left(t\right)}$ is the reset gate and $z^{\left(t\right)}$ is the update gate at time t; $W_{xr}$, $W_{hr}$, $W_{xz}$, $W_{hz}$ represent different weight parameters; $b_{r}$ and $b_{z}$ are the biases; the δ denotes logistics sigmoid function. Then, the hidden state $H^{\left(t\right)}$ at time t is computed as follows:

(4) $$C^{\left(t\right)}=tanh\left(W_{xc}X^{\left(t\right)}+W_{hc}\left(r^{\left(t\right)}⨀H^{\left(t-1\right)}\right)+b_{c}\right)$$

(5) $$H^{\left(t\right)}=z^{\left(t\right)}⨀H^{\left(t-1\right)}+\left(1-z^{\left(t\right)}\right)⨀C^{\left(t\right)}$$

where$\;C^{\left(t\right)}$ represents reset hidden states at time t; $W_{xc}$, $W_{hc}$ represent weight parameters; $b_{c}$ is the bias; the operator ⨀ refers to the Hadamard product of two matrices; tanh denotes hyperbolic tangent function. Then, the matrix multiplication in the GRU is replaced with the diffusion convolution to build the DCRNN model as follows:

(6) $$r^{\left(t\right)}=\delta \left(\Theta_{r}★B\left[X^{\left(t\right)},\;H^{\left(t-1\right)}\right]+b_{r}\right)$$

(7) $$z^{\left(t\right)}=\delta \left(\Theta_{z}★B\left[X^{\left(t\right)},H^{\left(t-1\right)}\right]+b_{z}\right)$$

(8) $$C^{\left(t\right)}=tanh\left(\Theta_{C}★B\left[X^{\left(t\right)},\left(r^{\left(t\right)}⨀H^{\left(t-1\right)}\right)\right]+b_{c}\right)$$

(9) $$H^{\left(t\right)}=z^{\left(t\right)}⨀H^{\left(t-1\right)}+\left(1-z^{\left(t\right)}\right)⨀C^{\left(t\right)}$$

where ★B represents the diffusion convolution as defined in Equation (1) and $\Theta_{r}$, $\Theta_{z}$, $\Theta_{C}$ are learnable parameters of the diffusion convolutional filters.

To perform multi-step air quality prediction, the DCRNN model utilizes the Sequence to Sequence (Seq2Seq) architecture, which is a typical Encoder-Decoder architecture based on RNN units [28,29]. In training the DCRNN model, we feed the input sequences (i.e., all the historical features $X\in {\mathbb{R}}^{I\times N}$) into the encoder and initialize the decoder using the final state of the encoder. Then, the decoder emits the predictions based on the observations. When testing the DCRNN model, we input the test set into the encoder and compare the corresponding prediction results generated by the decoder with the measured data to evaluate the proposed model. Generally, the DCRNN model can achieve accurate air quality predictions by simultaneously capturing the spatial dependencies of topological features among air quality monitoring network and temporal dependencies of multi-source inputs of air quality time series data. 

### 2.4. Graph Construction

Another important step of the DCRNN model is graph construction, which usually reflects the spatial relationships among geospatial data. In this paper, we map the air quality monitoring network with node and edge properties into one graph and calculate the weight matrix among edges over the graph. Generally, one element $w_{i,j}$ of the weight matrix is a reflection of the spatial correlation between nodes $v_{i}$ and $v_{j}$. Here, we use two types of graph construction methods: undirected and directed graphs. The construction of the undirected graph only considers the geographic distance between two monitoring stations as the spatial relationship:

(10) $$d_{ij}=d_{geo}\left(\left(x_{i},y_{i}\right),\left(x_{j},y_{j}\right)\right)$$

(11) $$W_{i,j}=\{\begin{array}{c} \exp\left(-\frac{d_{ij}^{2}}{\sigma^{2}}\right),d_{ij}<K\left(threshold\right)\\ 0,\;otherwise \end{array}$$

where ($x_{i},y_{i}$) represents the latitude and longitude coordinates of the node $v_{i}$, σ and K(threshold) are two user-defined hyperparameters.

**Figure 3:**
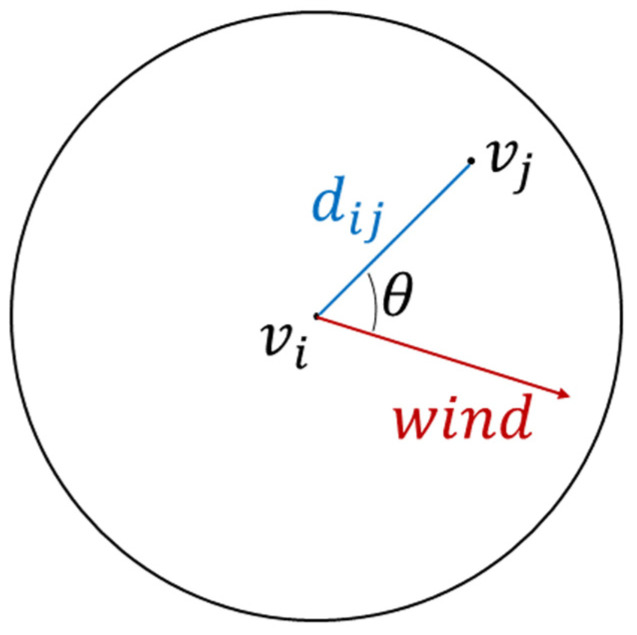
Spatial relationship between wind direction and directed graph construction. Wind vector shows the vector weighted average wind direction of each data group (i.e., Winter and Spring, Summer and Autumn).

In terms of the directed graph, we consider the effects of wind direction because wind direction is an important factor that greatly affects the dispersion of air pollutants, as revealed by previous studies [12]. In the undirected graph, $W_{i,j}$ is equal to $W_{j,i}$, but generally they are not equal in the directed graph due to the consideration of wind factors. Here, we use multiple transformations of the wind direction and geographic distance between nodes to construct different directed graphs and explore their influences on the prediction performances of the DCRNN model. Figure 3 illustrates the relationship between wind direction and directed graph construction, and Equations (12)–(16) clearly present five calculation methods of the edge weight matrix for the construction of different directed graphs as follows:

Directed graph 1:

(12) $$W_{i,j}=\{\begin{array}{c} \exp\left(-\frac{d_{ij}^{2}\sec^{2}\theta_{ij}}{\sigma^{2}}\right),-90{}^{\circ}\leq \theta_{ij}\leq 90{}^{\circ}\\ 0,\;otherwise \end{array}$$

Directed graph 2:

(13) $$W_{i,j}=\{\begin{array}{c} \exp\left(-\frac{d_{ij}^{2}\sin^{2}\theta_{ij}}{\sigma^{2}}\right),-90{}^{\circ}\leq \theta_{ij}\leq 90{}^{\circ}\\ 0,\;otherwise \end{array}$$

Directed graph 3:

(14) $$W_{i,j}=\{\begin{array}{c} \exp\left(-\frac{d_{ij}^{2}\left(1+\sin^{2}\theta_{ij}\right)}{\sigma^{2}}\right),-90{}^{\circ}\leq \theta_{ij}\leq 90{}^{\circ}\\ 0,\;otherwise \end{array}$$

Directed graph 4:

(15) $$W_{i,j}=\exp\left(-\frac{d_{ij}^{2}\left(2-\cos\theta_{ij}\right)}{\sigma^{2}}\right)$$

Directed graph 5:

(16) $$W_{i,j}=\{\begin{array}{c} \exp\left(-\frac{d_{ij}^{2}\sin\theta_{ij}}{\sigma^{2}}\right),-90{}^{\circ}\leq \theta_{ij}\leq 90{}^{\circ}\\ 0,\;otherwise \end{array}$$

### 2.5. Experimental Design

The experiment is conducted on a server with Ubuntu 16.04 Linux system, 128 GB memory, and NVIDIA Titan RTX (24GB GDDR5 VRAM) graphics card. Python 3.6, Pandas, NumPy, TensorFlow, and Keras are used for data processing and model configuration. In the experiment, the dataset is first divided into two groups according to the seasonal discrepancies for model construction and verification. Roughly 70%, 10%, and 20% of the dataset in each group are separately used for training, validating, and testing the proposed DCRNN model. Specifically, the training set and validation set are used to train the model and evaluate the model during the training process, respectively. The test set is only applied to provide an unbiased evaluation of a final model fit on the training set.

The loss function uses Mean Absolute Error (MAE) and the optimizer adopts adaptive moment estimation (Adam) to minimize the absolute error between the predicted and measured data. Hyperparameters are determined according to the model performances on the validation set. The early stopping technique is used for model training to improve training efficiency and avoid overfitting. Specifically, when the validation error cannot be further improved within the pre-specified number of cycles, the algorithm will terminate early, which can help reduce the computational costs. 

In this paper, different statistical indicators including MAE, Root Mean Squared Error (RMSE), and Pearson Correlation Coefficient (r) are used to evaluate the prediction performances of the proposed model, as computed below:

(17) $$\mathrm{MAE}=\frac{1}{n}\sum_{i=1}^{n}\left|O_{i}-P_{i}\right|$$

(18) $$\mathrm{RMSE}=\sqrt{\frac{1}{n}\sum_{i=1}^{n}{\left(O_{i}-P_{i}\right)}^{2}}$$

(19) $$r=\frac{\sum_{i=1}^{n}\left(O_{i}-{\bar{O}}_{i}\right)\left(P_{i}-{\bar{P}}_{i}\right)}{\sqrt{\sum_{i=1}^{n}{\left(O_{i}-{\bar{O}}_{i}\right)}^{2}}\sqrt{\sum_{i=1}^{n}{\left(P_{i}-{\bar{P}}_{i}\right)}^{2}}}$$

where $O_{i}$ and ${\bar{O}}_{i}$, respectively, refer to the observed values and their mean value, and $P_{i}$ and ${\bar{P}}_{i}$, separately, refer to the prediction value and their mean value.

## 3. Results and Discussion

### 3.1. Prediction Performances of DCRNN Using Different Graph Construction Methods

**Figure 4:**
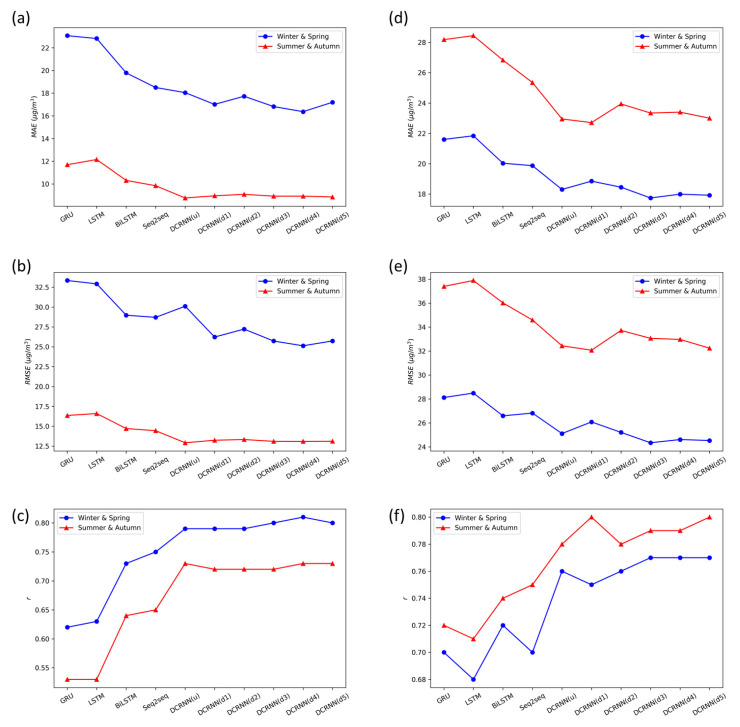
Model comparison between the DCRNN model and baseline models. (**a**) PM_2.5_, MAE; (**b**) PM_2.5_, RMSE; (**c**) PM_2.5_, r; (**d**) ozone, MAE; (**e**) ozone, RMSE; (**f**) ozone, r.

Table 1 and Table 2 present the prediction performances of the DCRNN models using different graph construction methods on the PM_2.5_ and ozone datasets, respectively. Figure 4 further illustrates the results of Table 1 and Table 2. Overall, the DCRNN models using any graph construction method all exhibit smaller error and higher precision for regional prediction of PM_2.5_ and ozone concentrations than other baseline models.

As seen in Figure 4a, the PM_2.5_ prediction results of the DCRNN models using directed graphs and undirected graphs both show smaller MAE than GRU, LSTM, bidirectional LSTM, and Seq2Seq models. Figure 4b indicates that the evaluation metric RMSE presents similar results with MAE in terms of model comparison. Furthermore, the MAE and RMSE from the winter and spring data group are both smaller for the directed graph model compared with the undirected graph model, particularly for the DCRNN models using directed graphs 3, 4, and 5 as shown in Equations (14)–(16). The result is partly attributed to the fact that the construction of directed graphs 3, 4, and 5 all consider the influences of geographic distance and wind direction on the variations in pollutant concentrations. By contrast, the prediction errors of the undirected graph model based on the summer and autumn data group are low enough so that the prediction performance of the directed graph model hardly shows extra superiority to the undirected graph model.

Similarly, the DCRNN models show less errors for regional prediction of ozone than the GRU, LSTM, bidirectional LSTM, and Seq2Seq models, as shown in Figure 4d,e. This result indicates that the DCRNN model can be well applied to regional prediction over a wide range of air pollutants, whether particulate matter or gaseous pollutants. The main difference between PM_2.5_ and ozone forecasts lies in that the performances of the directed graph model and the undirected graph model depend more on the dataset itself for ozone than PM_2.5_. Specifically, the directed graph models 3, 4, and 5 present smaller errors for the ozone forecasts on the winter and spring data group than the undirected graph model. However, the directed graph model does not perform significantly better than the undirected graph model on the summer and autumn data group. Overall, Figure 4f shows that directed graph model 5 exhibits the best agreement with the measured values among the DCRNN models with different graph construction methods.

In summary, the DCRNN model using directed graphs outperforms that using undirected graphs in the PM_2.5_ forecasts based on the winter and spring data group. There exist slight differences for the PM_2.5_ forecasts on the summer and autumn data group, as well as the ozone forecasts on the two data groups. However, the DCRNN model using directed graph 5 (Gauss Vector Weight), widely used in recent studies [12], generally brings the lowest prediction errors in both PM_2.5_ and ozone forecasts on all data groups. Therefore, we select the DCRNN model using directed graph 5 as the optimal directed graph model to conduct subsequent model comparison.

### 3.2. Multi Time-Step Prediction

Table 3 and Table 4 show the performances of the DCRNN model in different time-step predictions separately for PM_2.5_ and ozone. The prediction errors calculated from the directed graph and undirected graph models both demonstrate an increasing trend with a rise of the prediction time steps. The DCRNN model exhibits the smallest errors between the predicted and measured PM_2.5_ and ozone data at the 1st hour and shows the largest prediction errors at the 24th hour.

**Figure 5:**
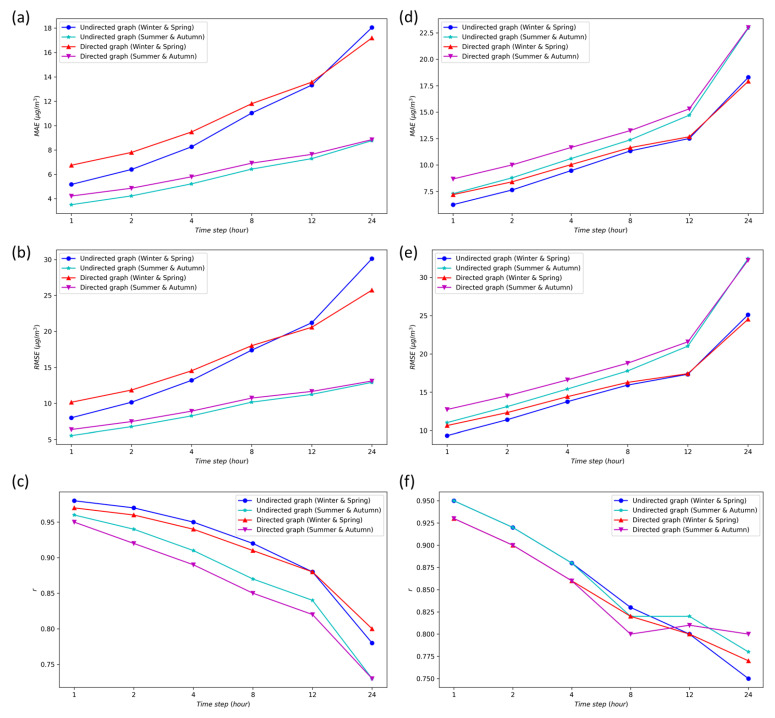
Regional prediction of PM_2.5_ and ozone based on the DCRNN model at different time steps. (**a**) PM_2.5_, MAE; (**b**) PM_2.5_, RMSE; (**c**) PM_2.5_, r; (**d**) ozone, MAE; (**e**) ozone, RMSE; (**f**) ozone, r.

Figure 5 provides a more intuitive visualization of the differences in model performances at different time steps. The DCRNN models using the undirected and directed graphs both show significant differences for the PM_2.5_ forecasts at different time steps. As shown in Figure 5a,b, the directed graph model on the winter and spring data group has smaller errors at long time steps above 12 h, while the undirected graph model has smaller errors at short time steps below 12 h. However, only when the time step is increased to 24 h, the directed graph model on the summer and autumn data group has similar performances to the undirected graph model.

Figure 5d,e illustrate that the ozone forecasts at different time steps present a similar pattern to the PM_2.5_ predictions. The directed graph model on the winter and spring data group performs better at long time steps beyond 12 h, while the undirected graph model performs better at short time steps below 12 h. In addition, the undirected graph model on the summer and autumn data group performs better at most time steps except for the 24 h time step. The above results suggest that the performance difference of the DCRNN models using undirected and directed graphs at different time steps could not be solely limited to the PM_2.5_ and ozone forecasts.

Overall, for short-term forecasts for the 1st to 8th hour, the DCRNN model using the undirected graph exhibits significantly smaller prediction errors than that using directed graphs. However, the discrepancy in prediction errors between the undirected graph and directed graph models gradually decreases with an increase in the prediction time steps. In contrast, for long-term forecasts in the next 12–24 h, the directed graph model has less prediction errors than the undirected graph model for the regional prediction of PM_2.5_ and ozone on the winter and spring data group. However, the directed graph model does not outperform the undirected graph model in the PM_2.5_ and ozone forecasts on the summer and autumn data group until the time step was increased beyond 24 h.

### 3.3. Spatial Distributions of PM_2.5_ and Ozone Forecasts based on the DCRNN Model

**Figure 6:**
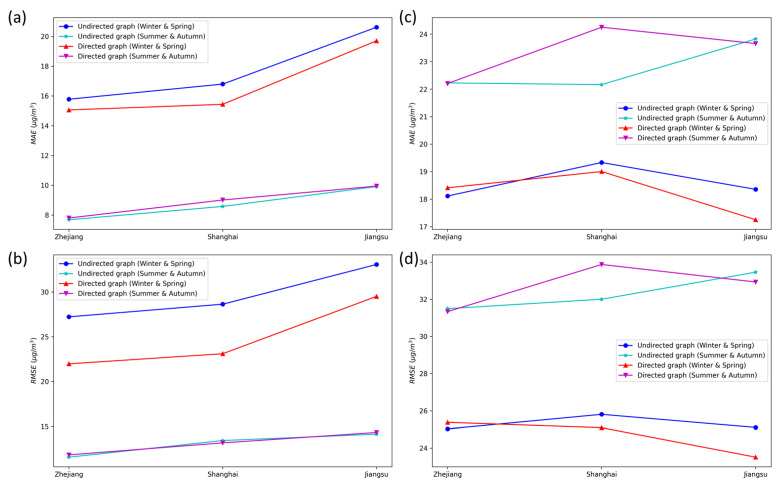
Model comparison between the DCRNN model and baseline models. (**a**) PM_2.5_, MAE; (**b**) PM_2.5_, RMSE; (**c**) ozone, MAE; (**d**) ozone, RMSE.

**Figure 7:**
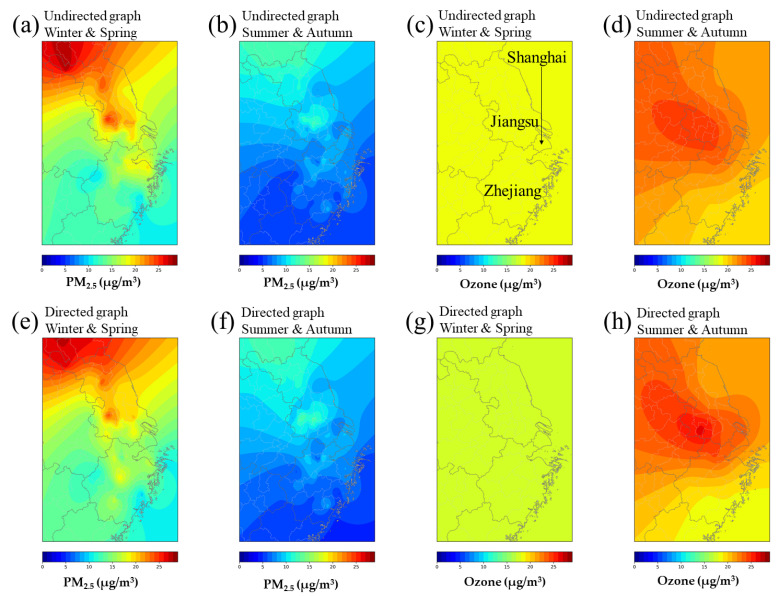
Spatial distributions of the mean MAE of 24 h air quality predictions based on the DCRNN model in the Yangtze River Delta region: (**a**) undirected graph, PM_2.5_ in winter and spring; (**b**) undirected graph, PM_2.5_ in summer and autumn; (**c**) undirected graph, ozone in winter and spring; (**d**) undirected graph, ozone in summer and autumn; (**e**) directed graph, PM_2.5_ in winter and spring; (**f**) directed graph, PM_2.5_ in summer and autumn; (**g**) directed graph, ozone in winter and spring; (**h**) directed graph, ozone in summer and autumn.

To further evaluate the spatial differences in the model performance, we divide the whole dataset into three groups according to the administrative provinces which the monitoring stations belong to, i.e., Shanghai, Zhejiang, and Jiangsu provinces. Table 5 and Table 6 respectively show the comparison of PM_2.5_ and ozone concentration prediction of the DCRNN model in the three provinces, and the above results are computed based on 24 h time steps. Figure 6 shows the visualization of the comparison of the above results. Figure 7 presents the results of the spatial distributions of MAE within the study area, using kriging interpolation to calculate the average MAE between predictions and observations in different geographical locations.

In terms of PM_2.5_ prediction, as shown in Table 5 and Figure 6, the DCRNN models based on directed and undirected graphs show a similar trend, with the lowest prediction error in Zhejiang Province, followed by Shanghai, and the largest prediction error in Jiangsu Province. In Figure 7a,b,e,f, the PM_2.5_ prediction errors of the undirected and directed graphs show the similar spatial distributions in the same seasons, but the northern province notably presents more prediction errors than the south. The larger prediction error in the northern province, i.e., Jiangsu province, could be related to two factors: (1) The relatively sparse distribution of air quality monitoring stations in the northern part of Jiangsu province increases the difficulty of providing refined data support for accurate air quality prediction. (2) Northern regions are usually accompanied by heavier particulate matter pollution and more variable PM_2.5_ concentrations, thus leading to larger PM_2.5_ forecast errors especially on heavily polluted days. 

In terms of ozone prediction, as shown in Table 6 and Figure 6, the ozone prediction errors of the directed graph model are smaller than that of the undirected graph model in Jiangsu province, which indicates that the ozone concentration variation in Jiangsu province could be closely related to wind factors and transport. In Shanghai, an opposite trend of ozone prediction error happens. The undirected graph model performs significantly better than the directed graph model for summer ozone concentration prediction. The results suggest that summer ozone pollution in Shanghai could be more related to local source emissions. In Figure 7c,d,g,h, the spatial difference of ozone prediction errors varies less than PM_2.5_ at the regional level, with smaller differences (MAE and RMSE) in Zhejiang, Shanghai, and Jiangsu provinces. Meanwhile, compared with the PM_2.5_ prediction results, the directed and undirected graph models both show stronger spatial variability in the ozone prediction errors.

## 4. Conclusions

In this study, we employ different construction methods of directed and undirected graphs to establish a novel diffusion convolutional recurrent neural network (DCRNN) model for the regional prediction of PM_2.5_ and ozone concentrations. The model can fully consider the spatial relationships between nodes within air quality monitoring network by integrating the combined effects of station-level geographic distance and dominant wind direction in the study area. Then, hourly PM_2.5_ and ozone data collected from 123 air quality monitoring stations in the Yangtze River Delta region are used to train, validate, and test the proposed DCRNN model. Several meaningful findings are summarized as follows:

(1) The DCRNN model outperforms the baseline models (e.g., GRU and LSTM) in PM_2.5_ and ozone forecasts.
(2) The DCRNN model using directed graphs with an integration of wind factors outperforms the undirected graph model in the long-term prediction of PM_2.5_ and ozone.
(3) The undirected graph model could achieve better performance in the short-term forecasts, particularly for the next 1st hour prediction.
(4) The prediction errors of the DCRNN model using undirected and directed graphs both suggest an upward trend with an increase in the prediction time steps, particularly for the undirected graph model.
(5) The monitoring stations that are sparsely distributed or located in heavily polluted areas could both cause lower prediction accuracy.

In terms of applications, the proposed model could assist environmental researchers in further improving the technologies of air quality prediction and serve as tools for environmental policymakers to implement related pollution control policies. The comparison results between the directed and undirected graph-based models for specific regions (e.g., provinces) and the inferences about the effects of wind factors on pollution in different regions could provide decision support for accurate pollution control.

One limitation of this study is that the DCRNN model restricts the dynamic characterization of the wind direction factor and just uses the weighted-average vector representing wind direction. In future studies, we could consider more advanced spatiotemporal prediction methods based on dynamic graph structures to model the dynamic effects of wind direction and further strengthen air quality prediction.

## Figures and Tables

**Table 1:** Model comparison between the DCRNN model and baseline models in the PM_2.5_ forecasts.

Model	Winter and Spring			Summer and Autumn		
	MAE (μg/m^3^)	RMSE (μg/m^3^)	r	MAE (μg/m^3^)	RMSE (μg/m^3^)	r
GRU	23.07	33.35	0.62	11.69	16.36	0.53
LSTM	22.82	32.93	0.63	12.15	16.60	0.53
Bidirectional LSTM	19.79	28.98	0.73	10.31	14.71	0.64
Seq2seq	18.50	28.71	0.75	9.84	14.44	0.65
DCRNN (undirected graph)	18.05	30.11	0.79	8.76	12.92	0.73
DCRNN (directed graph 1)	17.01	26.23	0.79	8.95	13.24	0.72
DCRNN (directed graph 2)	17.73	27.23	0.79	9.08	13.33	0.72
DCRNN (directed graph 3)	16.82	25.73	0.80	8.92	13.10	0.72
DCRNN (directed graph 4)	16.37	25.13	0.81	8.92	13.09	0.73
DCRNN (directed graph 5)	17.20	25.74	0.80	8.85	13.11	0.73

**Table 2:** Model comparison between the DCRNN model and baseline models in the ozone forecasts.

Model	Winter and Spring			Summer and Autumn		
	MAE (μg/m^3^)	RMSE (μg/m^3^)	r	MAE (μg/m^3^)	RMSE (μg/m^3^)	r
GRU	21.60	28.12	0.70	28.18	37.40	0.72
LSTM	21.84	28.49	0.68	28.44	37.89	0.71
Bidirectional LSTM	20.03	26.59	0.72	26.83	36.02	0.74
Seq2seq	19.87	26.82	0.70	25.35	34.59	0.75
DCRNN (undirected graph)	18.30	25.11	0.76	22.95	32.44	0.78
DCRNN (directed graph 1)	18.85	26.08	0.75	22.71	32.07	0.80
DCRNN (directed graph 2)	18.45	25.21	0.76	23.94	33.72	0.78
DCRNN (directed graph 3)	17.74	24.34	0.77	23.34	33.06	0.79
DCRNN (directed graph 4)	17.99	24.61	0.77	23.40	32.97	0.79
DCRNN (directed graph 5)	17.92	24.53	0.77	23.00	32.24	0.80

**Table 3:** The performance of the DCRNN model in forecasting PM_2.5_ concentrations at different time steps.

Data Group	Time-Step	Undirected Graph			Directed Graph		
		MAE (μg/m^3^)	RMSE (μg/m^3^)	r	MAE (μg/m^3^)	RMSE (μg/m^3^)	r
Winter and Spring	1 h	5.17	8.00	0.98	6.75	10.16	0.97
	2 h	6.40	10.16	0.97	7.80	11.85	0.96
	4 h	8.26	13.20	0.95	9.48	14.53	0.94
	8 h	11.03	17.40	0.92	11.80	18.02	0.91
	12 h	13.33	21.21	0.88	13.57	20.58	0.88
	24 h	18.05	30.11	0.78	17.20	25.74	0.80
Summer and Autumn	1 h	3.51	5.54	0.96	4.22	6.37	0.95
	2 h	4.23	6.77	0.94	4.86	7.47	0.92
	4 h	5.22	8.26	0.91	5.80	8.92	0.89
	8 h	6.43	10.17	0.87	6.92	10.74	0.85
	12 h	7.29	11.25	0.84	7.64	11.66	0.82
	24 h	8.77	12.92	0.73	8.85	13.11	0.73

**Table 4:** The performance of the DCRNN model in forecasting ozone concentrations at different time steps.

Data Group	Time-Step	Undirected Graph			Directed Graph		
		MAE (μg/m^3^)	RMSE (μg/m^3^)	r	MAE (μg/m^3^)	RMSE (μg/m^3^)	r
Winter and Spring	1 h	6.25	9.35	0.95	7.20	10.65	0.93
	2 h	7.64	11.41	0.92	8.41	12.34	0.90
	4 h	9.47	13.77	0.88	10.04	14.42	0.86
	8 h	11.33	15.93	0.83	11.64	16.28	0.82
	12 h	12.51	17.35	0.80	12.67	17.42	0.80
	24 h	18.30	25.11	0.75	17.92	24.53	0.77
Summer and Autumn	1 h	7.29	11.03	0.95	8.68	12.74	0.93
	2 h	8.79	13.12	0.92	10.01	14.53	0.90
	4 h	10.61	15.42	0.88	11.66	16.61	0.86
	8 h	12.38	17.78	0.82	13.25	18.79	0.80
	12 h	14.71	21.02	0.82	15.31	21.58	0.81
	24 h	22.95	32.44	0.78	23.00	32.24	0.80

**Table 5:** The performance of the DCRNN model in forecasting PM_2.5_ concentrations in different geographical areas.

Data Group	Province	Undirected Graph			Directed Graph		
		MAE (μg/m^3^)	RMSE (μg/m^3^)	r	MAE (μg/m^3^)	RMSE (μg/m^3^)	r
Winter and Spring	Shanghai	16.79	28.63	0.76	15.44	23.10	0.77
	Jiangsu	20.62	33.07	0.79	19.70	29.51	0.75
	Zhejiang	15.78	27.22	0.75	15.06	21.97	0.79
Summer and Autumn	Shanghai	8.58	13.43	0.78	9.01	13.17	0.79
	Jiangsu	9.91	14.13	0.72	9.94	14.33	0.71
	Zhejiang	7.68	11.67	0.72	7.80	11.83	0.71

**Table 6:** The performance of the DCRNN model in forecasting ozone concentrations in different geographical areas.

Data Group	Province	Undirected Graph			Directed Graph		
		MAE (μg/m^3^)	RMSE (μg/m^3^)	r	MAE (μg/m^3^)	RMSE (μg/m^3^)	r
Winter and Spring	Shanghai	19.33	25.81	0.69	19.00	25.09	0.72
	Jiangsu	18.36	25.10	0.75	17.25	23.51	0.79
	Zhejiang	18.11	25.03	0.76	18.41	25.38	0.76
Summer and Autumn	Shanghai	22.16	32.00	0.77	24.25	33.88	0.75
	Jiangsu	23.82	33.46	0.78	23.66	32.93	0.80
	Zhejiang	22.23	31.50	0.79	22.20	31.34	0.79

## Data Availability

The data presented in this study are available from the corresponding author on reasonable request.
